# Mitochondrial transport of protoporphyrinogen IX in erythroid cells

**DOI:** 10.18632/oncotarget.5124

**Published:** 2015-08-10

**Authors:** Yvette Y. Yien, Alessa R. Ringel, Barry H. Paw

**Affiliations:** Hematology Division, Brigham and Women's Hospital, Boston Children's Hospital and Harvard Medical School, Boston, MA, USA

**Keywords:** heme synthesis, porphyrin transporters, Tmem14c, iron and heme metabolism, erythropoiesis

Heme plays a vital role in essential processes such as detoxification, oxygen transport, circadian rhythm, microRNA processing, respiration, regulation of transcription and translation, and apoptosis. The majority of heme in the body is synthesized in red blood cells, whose function is to transport oxygen via the heme-containing oxygen carrier protein, hemoglobin. Defects in erythroid heme homeostasis can result in anemia, caused by the decrease in hemoglobin synthesis, porphyria, caused by accumulation of photoreactive heme intermediates, and iron overload [1].

**Figure 1:**
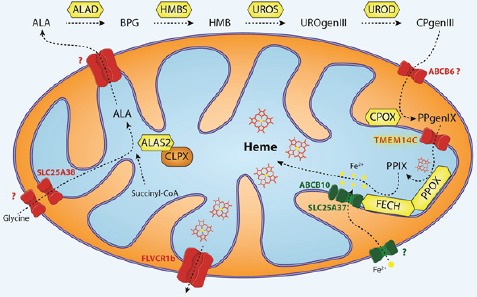
Intracellular trafficking of heme intermediates in erythroid cells Glycine is imported via SLC25A38 and condenses with succinyl-CoA to form δ-aminolevulinic acid (ALA) in a reaction catalyzed by ALA synthase (ALAS2 in red cells) [1]. ALAS is activated by mitochondrial chaperone ClpX through promoting the incorporation of pyridoxal phosphate, an essential cofactor for ALAS function [5]. After several catalytic conversions of heme precursors, coproporphyrinogen III (CPgenIII) is transported into the mitochondrial intermembrane space. It is then converted to protoporphyrinogen IX (PPgenIX) that is transported into the matrix by a mechanism requiring TMEM14C [3]. Ferrochelatase (FECH) metallates protoporphyrin IX (PPIX) with iron to form heme. In previous studies, we have shown that iron enters the mitochondrial matrix via mitoferrrin1 (SLC25A37) in the inner mitochondrial matrix. SLC25A37 is stabilized by ABCB10 and exists in a large oligomeric complex with FECH [6]. Heme is thought to be exported by FLVCR1b into the cytosol, where it is incorporated into hemoproteins [7]. Figure illustration courtesy of Johannes G. Wittig (Technische-Universität-Dresden, Germany).

Heme synthesis requires the coordinated transport of heme intermediates and iron within the cell and across membranes to provide substrates access to enzymes, prevent intercalation of photo-reactive heme intermediates into cellular membranes, and minimize generation of reactive oxygen species [1]. Most genetic studies of heme synthetic disorders, most commonly porphyria, have focused on mutations of heme synthesis enzymes. Disease symptoms are often dependent on environmental factors, indicating the presence of extragenic modifiers of the disease that participate in the heme synthesis pathway. Among potential modifiers are the genes required for the transport of heme, heme intermediates and iron (summarized in Figure 1). One such example is a loss-of-function mutation in *MFRN1 (SLC25A37)*, the erythroid mitochondrial iron transporter, which exacerbated protoporphyrin IX accumulation due to a gain-of-function C-terminal deletion in *ALAS2* [2].

In an RNAseq screen for transporters of heme intermediates in terminally differentiating erythroid cells, we identified *Tmem14c* as a gene that is required for heme synthesis and erythropoiesis in zebrafish and mice [3]. TMEM14C is an inner mitochondrial membrane protein with three tightly packed transmembrane helices, predictive of its function as a transporter [4]. It is required for the transport of protoporphyrinogen IX into the mitochondrial matrix, where it is converted to protoporphyrin IX and ultimately, heme. Strikingly, we observed coproporphyrin III accumulation and heme deficiency with normal expression of heme synthesis enzymes in *Tmem14c* deficient cells. This was the first published example of a porphyrin transport defect that caused porphyrin accumulation in the absence of additional mutations and suggests that *Tmem14c* is a potential modifier for anemias and porphyrias [3].

Up to E13.5, the development and viability of non-erythroid tissues were not compromised in *Tmem14c*-deficient mouse embryos [3]. As heme synthesis is also required for housekeeping processes such as mitochondrial respiration, our observations raise questions as to the identity of housekeeping protoporphyrinogen IX transporters. As *Tmem14c* is highly expressed in tissues that synthesize large amounts of heme, such as the adult liver, TMEM14C may play a porphyrin transport role in these tissues. In addition, TMEM14A, structurally similar to TMEM14C [4], may play an analogous role in protoporphyrinogen IX transport in housekeeping heme synthesis and partially compensate for the absence of *Tmem14c*, accounting for the normal appearance of *Tmem14c*-deficient embryos.

Conventional attempts to identify porphyrin transporters have proven challenging. Using complementary cell culture and animal models, we have identified a critical component of the erythroid porphyrin transport machinery. As heme synthesis in erythroid cells rely on specialized mechanisms to facilitate synthesis of large quantities of hemoglobin, a process not applicable to non-erythroid tissues, further studies are required to determine the identity of the protoporphyrinogen IX and other porphyrin transporters in non-erythroid cells. Our studies demonstrate that porphyrin transport proteins are critical regulators of heme synthesis and lay out a paradigm framework by which to identify other porphyrin transporters. These, and future studies will shed light on diseases resulting from defects in iron and heme metabolism, paving the way to successful design of therapeutic agents.
